# Book Reviews

**Published:** 1913-10

**Authors:** 


### BOOK REVIEWS.

---

*Books mentioned may be inspected at and ordered through this office. So far as possible, books received in any month will be reviewed in the issue of the second month following.*

---

MANUAL OF OBSTETRICS. John Osborn Polak, NM. S., M. D., Brooklyn, D. Appleton & Co., New York and London; 468 pages, 3 plates in color; 119 illustrations in text; limp leather; $3.00.

This treatise follows the golden mean between a condensed presentation of essentials and a cyclopædic monograph. Anatomy, embryology, physiology of pregnancy, abnormalities and clinical considerations of the pregnant state, physiology, mechanism and management of normal labor, the puerperium, the anatomy, physiology and management of the new born child, with a brief discussion of infant feeding, dentition, disease encountered early in life, pathology of labor and the puerperium, obstetric surgery, are the main topics. We are pleased at the retention of the time honored classification of the powers, passages and passenger, so aptly alliterative. Without in any sense implying a lack of authorship, we cannot refrain from complimenting the publishers upon the unusually elaborate mechanic preparation of this book.

---

ANATOMY, DESCRIPTIVE AND APPLIED, by Henry Gray, F. R. S.. New American Edition, revised and re-edited, by Edward Anthony Spitzka, M. D., Philadelphia. (Basle Anatomic Nomenclature in Latin, added to the ordinary). 1502 pages; 1225 engravings; Lea & Febiger, Philadelphia and New York; $6.00 cloth; $7.00 leather.

Gray's Anatomy, first appearing in England, half a century ago, united clear and accurate descriptions with illustrations superior to any previously published. An apparently minor point, the engraving of the names of structures directly upon their pictures, was, in itself, of the greatest convenience to students. While, even in a subject like anatomy, the science of medicine is never at a standstill, there is, obviously less opportunity for discovery and less need of modifying a treatise on this than in any other medical subject. Hence the retention of Gray's name is by no means an empty honor. The work is still essentially as he left it, although ample opportunity has been afforded for clarifying certain descriptions, for condensing others, tabulating structures according to the most ingenious methods devised by a multitude of teachers, for incorporating accrued experience in regard to anomalous disposition of organs, for revising the nomenclature, and, most important of all, for improving the illustrations in accordance with half a century of development of the engraver's art. There is an old story of a medical student who objected to studying anatomy from an old edition of his preceptor's library and who was rebuked by the latter with the sarcastic remark that very few new bones had been discovered since the book was printed. Such a masterpiece of medical publication as is here presented, justifies the student and reverses the original moral of the story. It is interesting to note that the present edition is the eighteenth in England and the nineteenth in the United States.

---

The Career of Dr. Weaver, A Novel. (By Mrs. Henry W. Backus. Cloth decorative; illustrated; 12 mo. pp. 373; net, $1.25; postpaid, $1.40, Boston; L. C. Page & Co.)

In a sense this is a "novel with a purpose." It contrasts the medical careers of two brothers, one a shrewd, ethical advertiser and fee splitter, successful socially, professionally and financially; the other, typic of the ordinary, sincere, honorable but not particularly prominent physician. Whether the purpose will be fulfilled to any degree by this story is an open question, but the very fact that a lay author has so thoroughly understood one of the most difficult problems that confront the profession and that this sort of success is an open book to the laity generally, ought to have some influence. Unfortunately, there are always some who will work the simplest and most often exposed trick and always a fresh crop of dupes. A quarter of a century ago one could scarcely go down town in Buffalo without encountering a young man, with whiskers, silk hat and funereal coat, waiting with his obstetric bag at the corner of Niagara and Main streets for a horse car that never came. During the Perry celebration, the same type, except that the clothes and whiskers were slightly modernized, the obstetric bag remaining the same, paraded through the crowd that was watching a procession which he did not take the trouble to look at. When we contemplate Dr. Weaver in his manifold phases, we are tempted to repeat the Pharisee's prayer of thankfulness that he was not as other men.

---

Topographic Maps of the U. S. Geologic Survey, issued by co-operation with the N. Y. State Engineer and Surveyor. During the last decade or more, the national government has joined with certain states in making accurate maps, showing water courses, roads, altitude, political divisions, roads and human habitation, etc. These maps are issued in quadrangles about ten miles wide and fifteen high (fifteen minutes of longitude and latitude, respectively). While the principal motive in preparing these maps has been to secure an accurate record of the physical geography of the country, they are of great interest to the medical profession as indicating drainage, potential water supplies, suitable altitudes and accessibility by highway and railroad, for sanitariums and the like. They will also probably be referred to in the future for historic data as to highways and settlements, perhaps to determine legal questions depending upon these matters. Military strategists will also probably use them considerably though, let us hope, only in retrospect, as the quadrangle including Gettysburg. Incidentally these maps are most convenient for those who tour the country in private conveyances, though requiring some further marking to indicate the present condition of roads. The work reflects great credit on the past and present force of the bureau.

---

University of Buffalo. Announcement of Courses In Arts and Sciences. Session of 1913-14. These courses are arranged in two semesters, extending from September 15 to June 5, the work being that of the freshman class in a standard college. Various items of interest with regard to recreation, students' organization, welfare, self-support, etc., are included. The officers and faculty of what we trust will develop into a collegiate department of the university are as follows:

The Council of the University has appointed as a committee to have supervision of these courses: Herbert U. Williams, M. D., Dean of the Department of Medicine; Willis G. Gregory, M. D., Dean of the Department of Pharmacy; Carlos C. Alden, J. D., Dean of the Department of Law; John Oppie McCall, D. D. S., of the Dental Department, Secretary-Treasurer.

Faculty—Albert P. Sy, M. S., Ph. D., Chemistry; Walter McM. Ralph, B. Chem., Chemistry; M. Smith Thomas, Physics; Lester B. Gary, A. B., Biology; Wilfred H. Sherk, A. M., Mathematics;

Peter Gow, Jr., A. B., Latin; Philip Becker Goetz, A. M., English; Julian Park, A. B., French; Wilhelm Oncken, German.

---

MARRIAGE AND GENETICS—Laws of Human Breeding and Applied Eugenics. By Charles A. L. Reed, M. D., F. C. S.;pp 182 (5¼x7¼); price, including postage, $1.00; subscription only. The Galton Press, Publishers, Cincinnati, Ohio.

The author speaks as a surgeon, impressed with duty to transmit a message that may prevent the disasters which so often culminate in the necessity for serious operations and which such operations can only partially mitigate, even in favorable cases. He quotes Galton's law of inheritance, which antedates and clearly anticipates that of Mendel, lacking, indeed, only its mathematic form of expression: (1) The germ cell is the summation of its racial antecedents derived in certain more or less definite proportions from each member of preceding ancestral generations. (2) On the average, as many heritable traits or characteristic are derived from one parent as from the other. We are naturally pleased to find that the author warmly supports the view which we have often expressed, that the study of genealogy is neither a waste of time, as most of those hold who have never given it attention, nor serviceable, as it must be confessed most professional and amateur genealogists have implied, merely as a matter of self-agrandisement or as a door-way admitting to organizations more or less worthy of encouragement. "The germ cell is so freighted with ancestral potentialities as to make the previous history of its germplasm through preceding generations a matter of deep concern to the conscientious prospective parent. This fact invests the subject of family trees and pedigrees with a new, definite and practical importance, particularly whenever the records embrace facts of character-determining significance to the progeny."

In the chapter on Genetic Factors, an alphabetic list is given of various drug habits, diseases, prominent symptoms, sexual and psychic abnormalities of significance in the medical examination preliminary to marriage. While not highly technical, this systematizes better than anything else of the sort that we have seen, the work of the physician as an advisor in regard to marriage. Inevitably in the present state of our knowledge regarding genetics, the book contains a good deal of trite generalization, including the Jukes-Edwards comparison. We have often felt like entering some other families in competition with the Edwards. It seems rather at variance with Galton's second law that the distinction of the Edwards descendants by the first wife is in such marked contrast with the mediocrity of the descendants by the second wife. Perhaps some of these descendants will speak for themselves later. At any rate, mediocrity is not a serious offense against society. However, we take personal pride in acknowledging the glowing tribute to our several times great aunt and wish that her brother might have been similarly gifted.

---

The Practical Medicine Series, Vol. 5. Pediatrics, edited by Isaac A. Abt; Orthopedic Surgery, edited by John Ridlon and Charles A. Parker. The Year Book Publishers, Chicago, 1913; 235 pages; illustrated; $1.35. (Price of series of ten volumes, $10.00.)

This is a comprehensive review of the literature on these two closely related branches of medicine, conforming to the excellent plan of the series. We are pleased to note references to Bartow of Buffalo and John R. Williams of Rochester.

---

A Compend on Bacteriology, Including Animal Parasites. Robert L. Pittsfield, Philadelphia. P. Blakiston's Son & Co., Philadelphia; 280 pages, four plates and 85 other illustrations, $1.00.

This is one of the familiar brown-cloth, Quiz Compends, which have done so much to fix medical teaching in the minds of students and which have been so often criticised for conforming to the practical requirements set by examiners. The author does not at all follow the plan of questions and answers, but presents briefly and succinctly and in analytic order, the prominent facts of bacteriology and parasitology. Whatever criticism is directed toward this method of teaching should, in all fairness, be reflected upon the system of examinations which have necessitated the method. And this system is defensible as representing the practically unanimous solution of how best to rate fitness for practice. Neither the system nor the Quiz method of teaching is ideal, but both represent the best method applicable to the average person, in medicine or any other science or art, that has thus far been evolved. A carefully prepared work like this has a field of usefulness that should not be overlooked. There are many physicians—80,000 or 100,000 in the United States—busily engaged in practice, with neither time, inclination nor technical skill adequate to pose as experts in bacteriology and utterly unable to follow the highly technical literature on this subject. The reviewer, for example, has a beautiful diploma, attested by the best authority, to the effect that he is a competent bacteriologist and pathologist. This diploma, measurably true when issued, with due regard to the rather rudimentary state of these sciences at that time, long ago became so untrue, that it was relegated to the attic. Yet all these physicians could better support true bacteriologists and could derive many practical lessons applicable to patients, if they possessed a modern knowledge of the general principles of bacteriology, of its technic and limitations. This work epitomizes the bacteriology and parasitology of today, and while admittedly falling far short of the requirements of the specialist in these branches, it is, for that reason, all the better adapted to the needs of the practitioner who wishes, not so much a handbook that will make him a bacteriologist—if such a book exists—as a compend which will give him an understanding of what the bacteriologist can do for him and how he can assist the laboratory expert in securing material for examination.

---

Malaria, Graham E. Henson, Jacksonville, Fla., with introduction by Charles C. Bass of New Orleans. C. V. Mosby Co., St. Louis; 190 pages; 27 illustrations; $2.50.

As is well known, the Mosby Co., publishers, by preference, monographs combining practical with thorough scientific study, so as to place in the reader's hands a work that is, so to say, the last if not the longest word on the subject essayed. No term better exemplifies the progress of medicine in the last generation than malaria. Once used as an inexact, comprehensive term for almost any undiagnosed condition, even including neurasthenia, it now has a most definite significance, supported by incontrovertible evidence of the most thorough nature and with a practical interpretation of scientific facts to apply to diagnosis and treatment, even to prophylaxis, which is, all things considered, excelled in the case of no disease and equaled in the case of only a very few others. For example, "Division, Protozoa; Class, Sporozoa; Order, Hæmosporidia; Genus, Plasmodium. Species: 1, Plasmodium malariæ, quartan; 2, Plasmodium vivax, tertian; 3, Plasmodium falciparum, æstivo-autumnal, tertian type; 4, Sub species, P. falciparum quotidianum, æstivo-autumnal, quotidian type. While it is possible that this zoologic classification may be amended in some particulars, it is a definite statement of relationship of a group of closely allied diseases, susceptible of microscopic demonstration and conforming to clinical observation.

The author discusses in detail not only the development of the various species and the methods of detection, but the epidemiology of the malarial fevers, their wide terrestrial distribution, the relative merits of quinine and other methods of medication—without upsetting the general belief in the almost exclusive indication for quinine in some form—the habits of the anopheles and the direct and indirect (drainage) methods of prophylaxis, as well as many interesting historic details and a detailed consideration of clinical manifestations. The author speaks from a wide experience and thorough study of the literature which warrant the expression of positive opinions. For example, he considers pernicious malaria as an aggravated form of any type, though usually of the æstivo-autumnal. He presents interesting and definite records of recurrence. We fail to note any reference to double forms, in particular to the theory that quotidian is a double tertian infection. Therapy is practically limited to quinine, but accurate tables are presented of the relative efficiency of various salts. Malaria is, fortunately, at present almost extinct in Buffalo, and, we believe, in most of the larger centers of population in the special field of this JOURNAL, but imported cases are liable to be encountered at any time, the anopheles has been found in various places and in smaller communities, physicians still have an active interest in malaria, which was formerly prevalent throughout this territory.

